# Acknowledgment to Reviewers of *Diseases* in 2020

**DOI:** 10.3390/diseases9010011

**Published:** 2021-01-27

**Authors:** Diseases Editorial Office

**Affiliations:** MDPI AG, St. Alban-Anlage 66, 4052 Basel, Switzerland

Peer review is the driving force of journal development, and reviewers are gatekeepers who ensure that *Diseases* maintains its standards for the high quality of its published papers. Thanks to the cooperation of our reviewers, in 2020, the median time to first decision was 17.6 days and the median time to publication was 35.5 days. The editors would like to express their sincere gratitude to the following reviewers for their precious time and dedication, regardless of whether the papers were finally published:
Adey, DeborahDec, MartaAi, YongDey, PriyankarAkil, LumaDi Micco, PierpaoloAnnunziata, GiuseppeEfird, Jimmy T.Asakawa, MitsuhikoErdmann, KatiAtehortua, Nelson A. De La PenaEscolà-Gil, Joan CarlesAyala-Zavala, J. F.Faita, FrancescoBerndt, Michael C.Fiorucci, StefanoBhuiyan, AzadFonseca, KevinBondy, Stephen C.Franzoni, FerdinandoBresciani, ElenaFujita, ToshitsuguBrieño-Enriquez, MiguelFurmańczyk, KonradBrown, MarkGalassi, AndreaBuchet, ReneGao, BeiByeon, HaewonGarinis, GeorgeCappello, FrancescoGazzonis, Alessia LiberaCareem, FaizalGiacobini, MaiBrittCarlini, Célia R.Giesbrecht, Gerald F.Casella, GiovanniGobba, Fabrizio MariaCastiglia, PaoloGordon, Catherine A.Chatzikyrkou, ChristosGuerini, Franca R.Chen, GuoxunGuerriero, GiuliaChen, Tai-HengGuetl, KatharinaCheng, Hung-ChiGutenbrunner, ChristophChowdhury, Imran HussainHamaguchi, MasahideChreptowicz, KarolinaHanyaloglu, Aylin C.Conn, David BruceHayashida, KyokoConti, MicheleHe, Jin-ShengCretoiu, DragosHeleniak, ZbigniewCunningham, LisaHiebert, PaulDampier, CarltonHinds, RupertDario, BrunettiHolt, StephenDe Spiegelaere, WardHsu, LewisDe Villiers, MarianneHu, AndyDean, FrankHu, XiaoyuDeb, SubrataHuang, Helen J.Huang, KaiPace, FabioIsmail, Hanafy MahmoudPacenti, MoniaJunichi, KitanakaPalomba, LetiziaKadotani, HiroshiPalomino-Moral, Pedro ÁngelKandeel, MahmoudPande Katare, DeepshikhaKane, EleanorPasaje, Charisse Flerida A.Kashfi, KhosrowPasquinelli, GianandreaKato, Takamitsu APatel, DevenKeenan, Jeffrey A.Pence, BrandtKenmochi, TakashiPérez-García, SeleneKim, KaPhalak, PoonamKim, Won YoungPiana, AndreaKlinger, MarianPleass, HenryKołodziejczak, MichalinaPrado-Cabrero, AlfonsoKonhilas, JohnRathinasabapathy, AnandharajanKouji, HaradaRavaioli, StefanoKrintus, MagdalenaReynolds, MaryKuryłowicz, AlinaRibaldone, DavideLamichhane, SantoshRomine, WilliamLau, Wei LingRosado, CarlosLazarevic-Pasti, TamaraRosenthal, KenLi, BinxingRoubertoux, PierreLi, TieshiScapellato, Maria LuisaLichtenauer, MichaelSchermer, Julie AitkenLin, Cheng-WenSergi, Pier NicolaLin, Pei-HuiSergunin, AlexanderLiu, ShichaoSharma, IshaLu, JunSinigaglia, AlessandroMaddelein, Marie-LiseSnelling, WilliamMarcoulides, George A.Sorahan, TomMartín Casanueva, Miguel AngelSpeeckaert, MarijnMascellino, Maria TeresaSplichal, IgorMastrangelo, StefanoSridhar, SubbaramiahMaugeri, Andrea GiuseppeStack, GaryMeerson, AriStieger, BrunoMesch, GustavoSun, ChongkuiMetzinger, LaurentSuzuki, TohruMizuno, MasashiTajiri, YujiMlocicki, DanielTamayo-Ortiz, MarcelaModesti, AlessandraTavoschi, LaraMoles, LauraTeschke, RolfMoret-Tatay, CarmenThomas, RogerMuniyan, SakthivelTimson, Benjamin F.Narayan, PremaToietta, GabrieleNastasi, ClaudiaTomsone, SigneO’Hara, StevenTonetti, LorenzoObermüller, NicholasTripathi, ManishOkech, BernardTünde, TarrOsuna, EduardoUsai Satta, PaoloOwino, Simon OderaVaandrager, Arie B.Venusia, CovelliWang, XiangruVeraldi, StefanoWei, ChengguoVezzoli, ValeriaXu, Wen TaoVigilato, Marco Antonio NatalYang, Jiann-JouVukojevic, KatarinaYeligar, Samantha M.Wang, Chih-HaoZhang, ChengxianWang, CuicuiZhang, Duo

